# Domestic violence among antenatal attendees in a Kathmandu hospital and its associated factors: a cross-sectional study

**DOI:** 10.1186/s12884-016-1166-7

**Published:** 2016-11-21

**Authors:** Monika Shrestha, Sumina Shrestha, Binjwala Shrestha

**Affiliations:** Department of Community Medicine and Public Health, Institute of Medicine, Tribhuvan University, Kathmandu, Nepal

**Keywords:** Domestic violence, Violence, Pregnant women, Antenatal care, Factors associated

## Abstract

**Background:**

Domestic violence during pregnancy is a public health problem which violates human rights and causes an adverse effect on both maternal and fetal health. The objectives of the study were to assess the prevalence of domestic violence among the pregnant women attending the antenatal clinic, to explore the associated factors, and to identify the perpetrators of domestic violence.

**Methods:**

A descriptive cross-sectional study was conducted among 404 pregnant women in their third trimester of pregnancy. Convenient sampling was used to select the study population. Data collection tools consisted of questionnaires on socio-demographic characteristics of the woman and her spouse, social support, and the woman’s attitude towards domestic violence, along with her experiences of psychological, physical, and sexual violence. Domestic violence was assessed using a questionnaire adapted from a World Health Organization multi-country study on women’s health and life experiences. Relationships between domestic violence and the various factors were determined by bivariate analysis using a chi-square test. Binary logistic regression with 95% confidence interval and adjusted odds ratio were then applied to assess the factors independently associated with domestic violence.

**Results:**

More than one-quarter (27.2%) of the pregnant women had experienced some form of violence. The most common form of violence was sexual violence (17.3%), followed by psychological violence (16.6%) and physical violence (3.2%). Husbands within the age group 25–34 years (AOR = 0.38), women married for 2–5 years (AOR = 0.42) and who had one or two children (AOR = 0.32) were negatively associated with domestic violence. Whereas the presence of husband’s controlling behavior (AOR = 1.88) and experience of violence before the current pregnancy (AOR = 24.55) increased the odds of experiencing violence during pregnancy. The husband was the major perpetrator in all type of violence.

**Conclusions:**

Domestic violence is common among pregnant women attending an antenatal clinic. It indicates a need for routine screening during antenatal visits to identify women experiencing violence and thus provide support services, thereby preventing them from adverse health consequences.

## Background

Violence against women has been recognized globally as the most pervasive public health problem violating human rights and causing substantial social, economic and health problems [1]. According to World Health Organization (WHO), domestic violence (DV) is defined as psychological/emotional, physical or sexual violence or threats of physical or sexual violence that are inflicted on a woman by a family member: an intimate male partner, marital/cohabiting partner, parents, siblings, or a person very well known within the family or a significant other (i.e., former partner) when such violence often takes place in the home [2]. A meta-analysis of 92 independent studies concerning DV among pregnant women showed an average prevalence of emotional abuse of 28.4%, and prevalence rates of physical abuse and sexual abuse were 13.8 and 8.0%, respectively [3].

Most of the violence are perpetrated by a woman’s intimate male partner than from any other perpetrators [4]. The prevalence of intimate partner violence during pregnancy in a study conducted in 19 countries varied from 2.0 to 13.5% [5]. Violence during pregnancy ranged between 4.3 and 48% in a study conducted in some of the Asian countries [6]. It has been observed that the prevalence of DV during pregnancy in less developed countries is higher (27.7%) than that in developed countries (13.3%) [3]. Although there is a growing evidence on the magnitude, underlying factors, and adverse outcomes of the problem, most studies originate from the developed countries [7].

Violence against women has an overwhelming effect on both women’s sexual and reproductive health, as well as on the health of their children [8]. Violence during pregnancy is associated with obstetric problems, premature rupture of membranes, urinary tract infections, vaginal bleeding, lack of sexual desire [9], depressive symptoms [10] and antepartum hemorrhages [11] in women. Similarly, studies have also reported that violence is significantly associated with adverse maternal health behavior including drinking during pregnancy, and late prenatal care [12]. Violence is also associated with an increased risk of intrauterine growth restriction, perinatal death [11], preterm delivery, stillbirth, miscarriage [13], and low birth weight [14, 15]. Studies have also found associations between Intimate partner violence (IPV) and behavioral risk factors such as alcohol and drug abuse [15].

Although efforts are being made to address the violence towards women of reproductive age, there have been few studies focusing on DV during pregnancy in Nepal. Nepal Demographic Health Survey (NDHS, 2011) indicated that 6% of women who have been pregnant experienced physical violence during their pregnancy, though this did not take into account other forms of violence. Thus, the findings from our study are expected to improve the current understanding of DV during pregnancy and to facilitate the appropriate planning of policies and programs in addressing DV during pregnancy. The objectives of the study were to assess the rate of DV during pregnancy in a sample of pregnant women attending antenatal care clinic at Tribhuvan University Teaching Hospital (TUTH), to explore the associated factors, and to identify the perpetrators of DV.

## Methods

### Study design and site

A descriptive cross-sectional study was conducted in TUTH. TUTH is one of the centrally located tertiary level health care facilities in Kathmandu, the capital city of Nepal.

### Study population

The study population comprised of pregnant women coming to TUTH for their antenatal check-up. Women in the third trimester of the pregnancy and those who had been living with any of the family members from the beginning of the conception were included in the study.

### Sampling technique and sample size

Convenient sampling was used to interview the pregnant women. Only pregnant women who were eligible and willing to participate were included in the study. The required sample size for the study was calculated using Epi Info 7 taking a 90% power and 95% confidence interval (CI). Taking into account the 5% non-response rate, the total sample size interviewed was 404. Prevalence of DV among pregnant women in Nepal was assumed to be 50%.

### Data collection tools and techniques

The data was collected by the principal investigator as well as three trained female data collectors from 16^th^ September to 11^th^ November 2015 by conducting face to face interviews with the pregnant women. The researchers were thoroughly informed about the study and the ethical issues involved prior to the data collection. Only those eligible pregnant women who agreed to participate were included in the study. The questionnaires for the study were developed mainly by adapting questions from the WHO multi-country study on women’s health and life experiences (2005) and also from other relevant studies. The semi-structured questionnaires were used for the data collection, and the interview included questions on socio-demographic characteristics of the woman and her spouse, social support and the woman’s attitude on DV, along with her experiences of psychological, physical and sexual violence. The questionnaires were drafted in English and then translated into the native language (Nepali). Pretesting of the questionnaire in Nepali among the non-sampled pregnant women was carried out. After pretesting of the tool, necessary changes in the questionnaire were made before the actual data collection.

## Study variables

### Measure of dependent variable

DV during the current pregnancy was used as a dependent variable for the study. The pregnant women were asked if they had experienced one or more acts of psychological, physical or sexual violence within or outside the home during their current pregnancy. The acts included to measure various forms of violence were:

Psychological violence - insulted or made her feel bad about herself, said or did something to humiliate her in front of others, threatened to hurt/harm her or someone close to her, and scared or intimidated her on purpose.

Physical violence - slapped, pushed, shoved or beat her, twisted arm or hair or kicked her, threatened or actually used a knife or other weapon, punched or hit her with something that could hurt her, choked her, burned or scalded her on purpose, and punched or kicked in the abdomen.

Sexual violence - insisted on having sexual intercourse even when she did not want to but did not use physical force, physically forced her to have sexual intercourse even when she did not want to, and insisted her to do any sexual act that she felt to be degrading and humiliating.

Those women who reported at least one of the three forms of the violence were considered to have experienced DV.

### Measure of independent variables

Independent variables are divided into three categories: *first*, socio-demographic characteristics of the women (age, ethnicity, religion, education, occupation, type and duration of marriage, type of family, family size, economic status, number of living children, intended pregnancy, abortion, and experience of violence before); *second*, characteristics of the husbands (age, education, occupation, alcohol consumption, extramarital relationships and husband’s controlling behaviour); and *third*, social support (natal family or friend for help/support and member of any community group/organization) and the woman’s attitude on domestic violence (attitude towards wife beating and attitude in refusal of sex).

Husbands’ controlling behaviour included one or more of the following acts towards women by their husband: a) keeps her from seeing friends; b) restricts her contact with her family; c) insists on knowing where she is at all times; d) is jealous or gets angry if she talks to other men; e) frequently accuses her of being unfaithful; f) expects to ask permission before seeking health care for herself. If the presence of any of the above six acts was reported by women, then the presence of husband’s controlling behavior was said to be present.

Also, women were asked under which of the following circumstances they believe it is considered acceptable for a man to hit or physically mistreat his wife. The acts included: a) if she does not complete her household work to his satisfaction; b) if she disobeys him; c) if she argues with him; d) if she disrespects her in-laws; e) if she goes out without permission f) if she refuses to have sex with him; g) if he finds out that she has been unfaithful. The responses were categorized as not accepting to any of the above acts, partially accepting (1–3 acts) and highly accepting (4–7 acts).

Likewise, women were asked if they could refuse to have sex with her husband under the given circumstances: a) if she does not want to; b) if he is drunk; c) if he mistreats her. The responses were categorized as: completely refuse (in all matters) and does not refuse at all or partially refuse (1–2 matters).

### Data processing and analysis

The data were first coded and entered in EpiData (version 3.1). After importing the entered data into SPSS (version 17), data checking, cleaning, and recoding were performed for further analysis. Bivariate analysis was done using the chi-square test to investigate the association between DV during pregnancy and the independent variables. Multivariate analysis was carried out using binary logistic regression for those variables which were significant (*p* < 0.05) at 95% CI in the bivariate analysis after checking for multicollinearity [Variance Inflation Factor (VIF) <10]. Odds ratio (OR) and adjusted odds ratio (AOR) at 95% was calculated to determine the strength of the relationship between dependent and independent variables.

## Results

### Characteristics of the study population

The mean age of the pregnant women was 25.5 years (±4.3) and approximately two-fifths (43.8%) of them belonged to age group of less than 25 years. Three-fifths of the women belonged to the upper caste group (61.1%). Hindus accounted more than four-fifths (86.2%) of the study population. Most of the women were literate (96.3%), and more than three-fifths (64.9%) of them were unemployed. More than half (57.9%) of the women were in an arranged marriage. The mean duration of marriage was 4.15 (±3.76) years with approximately 71% of the women married for less than of 5 years. Most of the women lived in a joint/extended family and three-quarters of the women lived in a family of fewer than six members. Three out of five women had only one child (63.9%). More than three-quarters (77.2%) of the women reported that their pregnancy was planned and had no history of abortion/miscarriage. One-fifth of the women experienced violence before pregnancy (Table 1).

**Table 1:** Descriptive characteristics of pregnant women and its association with domestic violence during current pregnancy

Characteristics	Number = 404 (%)	Experience of domestic violence		*p* value	Crude OR
		No	Yes		
Age of woman					
< 25	177 (43.8)	130 (44.2)	47 (42.7)		Ref
25–29	149 (36.9)	108 (36.7)	41 (37.3)	0.845	1.05 (0.64–1.72)
≥ 30	78 (19.3)	56 (19)	22 (20)	0.784	1.09 (0.60–1.97)
Ethnicity					
Upper caste	247 (61.1)	192 (65.3)	55 (50)		Ref
Others^a^	157 (38.9)	102 (34.7)	55 (50)	0.005*	1.88 (1.21–2.94)
Religion					
Hindu	348 (86.2)	260 (88.4)	88 (80)		Ref
Buddhist/Christian/Kirat/Muslim	56 (13.8)	34 (11.6)	22 (20)	0.031*	1.91 (1.06–3.44)
Education of woman					
Literate	389 (96.3)	285 (96.9)	104 (94.5)		Ref
Illiterate	15 (3.7)	9 (3.1)	6 (5.5)	0.264	0.55 (0.19–1.58)
Occupation of woman					
Unemployed	262 (64.9)	192 (65.3)	70 (63.6)		Ref
Employed	142 (35.1)	102 (32.7)	40 (36.4)	0.754	1.08 (0.68–1.70)
Type of marriage					
Arrange marriage	234 (57.9)	179 (60.9)	55 (50.0)		Ref
Love marriage	170 (42.1)	115 (39.1)	55 (50.0)	0.049*	1.56 (1.00–2.42)
Duration of marriage					
≤ 1	142 (35.1)	91 (31)	51 (46.4)		Ref
2–5	146 (36.1)	117 (39.8)	29 (26.4)	0.003*	0.44 (0.26–0.75)
6–9	87 (21.6)	67 (22.8)	20 (18.2)	0.042*	0.53 (0.29–0.98)
≥ 10	29 (7.2)	19 (6.5)	10 (9.1)	0.883	0.94 (0.41–2.17)
Type of family					
Nuclear	153 (37.8)	105 (35.7)	48 (43.6)		Ref
Joint/Extended	251 (62.2)	189 (64.3)	62 (56.4)	0.145	0.72 (0.46–1.12)
Family size					
≤ 5 members	301 (74.5)	218 (74.1)	83 (75.5)		Ref
> 5 members	103 (25.5)	76 (25.9)	27 (24.5)	0.789	0.93 (0.56–1.55)
Economic Status					
Rich	–	159 (54.1)	43 (39.1)		Ref
Poor	–	135 (45.9)	67 (60.9)	0.008*	1.84 (1.17–2.87)
Number of living children					
None	258 (63.9)	178 (60.5)	80 (72.7)		Ref
One or two children	146 (36.1)	116 (39.5)	30 (27.3)	0.024*	0.58 (0.36–0.93)
Intended pregnancy					
No	92 (22.8)	59 (20.1)	33 (30)		Ref
Yes	312 (77.2)	235 (79.9)	77 (70)	0.035*	0.59 (0.36–0.96)
Abortion/Miscarriage					
No	302 (74.8)	227 (77.2)	75 (68.2)		Ref
yes	102 (25.2)	67 (22.8)	35 (31.8)	0.064	1.58 (0.97–2.57)
Experience of violence before					
No	323 (80.0)	275 (93.5)	48 (43.6)		Ref
Yes	81 (20.0)	19 (6.5)	62 (56.4)	<0.001*	18.70 (10.28–34.01)

*Ref* = Reference

* = *p* value <0.05

^a^Disadvantaged, advantaged janajatis, dalit, disadvantaged non dalit terai people, religious minorities

The majority of the respondents’ husbands (70%) belonged to the age group of 25–34 years, and almost all of them were literate (97.8%) and employed (96.5%). Slightly more than half of the spouses consumed alcohol (53.5%). Around 90% of the pregnant women reported that their husband did not have an affair with another woman and slightly less than half of them stated to have experienced at least one of the six controlling behavior from their husband (Table 2).

**Table 2:** Characteristics of pregnant women’s husband and its association with domestic violence during current pregnancy

Characteristics	Number = 404 (%)	Experience of domestic violence		*p* value	Crude OR (95% CI)
		No	Yes		
Age of husband					
< 25	55 (13.6)	28 (9.5)	27 (24.5)		Ref
25–34	283 (70.1)	218 (74.1)	65 (59.1)	<0.001*	0.31 (0.17–0.56)
≥ 35	66 (16.3)	48 (16.3)	18 (16.4)	0.014*	0.39 (0.18–0.83)
Education of husband					
Literate	395 (97.8)	291 (99.0)	104 (94.5)		Ref
Illiterate	9 (2.2)	3 (1.0)	6 (5.5)	0.016*	5.60 (1.38–22.78)
Occupation of husband					
Unemployed	14 (3.5)	11 (3.7)	3 (2.7)		Ref
Employed	390 (96.5)	283 (96.3)	107 (97.3)	0.621	1.39 (0.38–5.07)
Alcohol consumption by husband					
No	188 (46.5)	149 (50.7)	39 (35.5)		Ref
Yes	216 (53.5)	145 (49.3)	71 (64.5)	0.007*	1.87 (1.19–2.94)
Extramarital relationship of husband					
No	365 (90.3)	273 (92.9)	92 (83.6)		Ref
Yes	14 (3.5)	3 (1.0)	11 (10.0)	<0.001*	10.88 (2.97–39.86)
Don’t know	25 (6.2)	18 (6.1)	7 (6.4)	0.756	1.15 (0.47–2.85)
Husband’s controlling behaviour					
No	206 (51.0)	172 (58.5)	34 (30.9)		Ref
Yes	198 (49.0)	122 (41.5)	76 (69.1)	<0.001*	3.15 (1.98–5.02)

*Ref* = Reference

* = *p* value <0.05

Many women expressed that they had a natal family (89.6%) and friends (71.0%) for help or support when needed. About two-fifths (22.3%) of the women were members of a community group or organization. Approximately one-half (47.8%) of women had a partially accepting attitude on men justified to beat their wives, and most of the women (95.3%) expressed that the women could refuse the demand of sex on all three matters (Table 3).

**Table 3:** Social support and women’s attitude on domestic violence related characteristics and its association with domestic violence during current pregnancy

Characteristics	Number = 404 (%)	Experience of domestic violence		*p* value	Crude OR (95% CI)
		No	Yes		
Natal family for help/support					
Yes	362 (89.6)	260 (88.4)	102 (92.7)		Ref
No	42 (10.4)	34 (11.6)	8 (7.3)	0.212	0.60 (0.27–1.34)
Friend for help/support					
Yes	287 (71.0)	220 (74.8)	67 (60.9)		Ref
No	117 (29)	74 (25.2)	43 (39.1)	0.006*	1.91 (1.20–3.04)
Member of any community group/organization					
Yes	90 (22.3)	74 (25.2)	16 (14.5)		Ref
No	314 (77.7)	220 (74.8)	94 (85.5)	0.024*	1.98 (1.09–3.57)
Women’s attitude towards wife beating					
Not accepting	187 (46.3)	149 (50.7)	38 (34.5)		Ref
Partially accepting (1–3)	193 (47.8)	132 (44.9)	61 (55.5)	0.013*	1.81 (1.14–2.89)
Highly accepting (4–7)	24 (5.9)	13 (4.4)	11 (10.0)	0.007*	3.32 (1.38–7.99)
Women’s attitude in refusal of sex					
Completely refuse (3 matters)	385 (95.3)	284 (96.6)	101 (91.8)		Ref
Doesn’t refuse at all/Partially refuse (1–2 matters)	19 (4.7)	10 (3.4)	9 (8.2)	0.062	2.53 (1.00–6.41)

*Ref* = Reference

* = *p* value <0.05

### Prevalence and types of violence

More than one-quarter (27.2%, 95% CI: 27.16%–27.24%) of the pregnant women were found to experience some form of DV from different perpetrators. The most common form of violence among the three types was sexual violence, which accounted for 17.3% (95% CI: 17.26%–17.34%) of the cases. Psychological violence was experienced by 16.6% (95% CI: 16.56%–16.64%) of the pregnant women, and 3.2% (95% CI: 3.18%–3.22%) of the women were found to experience physical violence (Table 4).

**Table 4:** Prevalence of domestic violence against pregnant women according to type of violence

Forms of violence	Number = 404	Percentage (%)
Psychological violence^a^		
Insulted/made feel bad about self	53	13.1
Said or did something to humiliate in front of others	16	4
Scared or intimidated on purpose	20	5
Threatened to hurt/harm you or someone close to you	3	0.7
At least one episode of psychological violence	67	16.6 ± 0.04
Physical violence^a^		
Slapped/pushed/shoved/beat	19	4.7
Twisted arm or hair/kicked	4	1.0
Punched or hit/threw something that could hurt you	3	0.7
Choked you	1	0.2
At least one episode of physical violence^a^	13	3.2 ± 0.02
Sexual violence		
Insisted to have sexual intercourse (but did not use physical force)	70	17.3
Physically forced to have sexual intercourse	2	0.5
Insisted to do any sexual act that felt to be degrading or humiliating (but did not use physical force)	1	0.2
At least one episode of sexual violence	70	17.3 ± 0.04
Psychological, physical or sexual violence (at least any one episode of three violence)	110	27.2 ± 0.04

^a^Multiple responses

### Perpetrator of violence

The husband was the main perpetrator in all types of violence. Among those who experienced psychological violence, more than half (65.6%) of them reported that their husband was the major perpetrator, followed by their mother-in-law who contributed to 19.4% of the violence and sister-in-law accounting for 9% of the cases. The other perpetrators were father-in-law and brother-in-law, both of whom accounted to contribute 2 cases each. Regarding both physical and sexual violence, the sole perpetrator was the husband.

### Association of domestic violence and women’s socio-demographic factors

The result of the bivariate analysis between DV during pregnancy and socio-demographic characteristics of women are presented in Table 1. Women belonging to other ethnicities were significantly more likely to experience DV than those of the upper caste. The women who followed Buddhism, Christianity or Kirat, were at higher risk to be abused during the pregnancy as compared to women who were Hindu. Furthermore, the women who had love marriage were approximately two times more likely to experience DV than who were part of an arranged marriage. Compared to women married for less than and equal to 1 year, women married for 2–5 years and 6–9 years were both less likely to report DV. Likewise, the odds of reporting violence during pregnancy were two times higher among poor women. Women bearing one or two children and those who had a history of abortion/miscarriage were less likely to experience DV. Also, women whose pregnancies were intended had less chance of reporting DV. In addition, women who had a history of the experience of DV before pregnancy were 18 times more likely to have been exposed to violence during pregnancy.

### Association of domestic violence in pregnant women and husbands’ characteristics

Table 2 shows a clear association between DV and husbands’ characteristics such as age, education, alcohol consumption, extramarital relationship, and controlling behavior of the husband. The husbands belonging to the age group of 25–34 years and 35 and above were both significantly negatively associated with DV. Likewise, women whose husbands were illiterate were more likely to experience DV. Alcohol consumption by husbands was also found to be positively associated with DV and those women whose husbands had extramarital relationships and who controlled them were more likely to report violence.

### Association of domestic violence and women’s social support and attitude towards domestic violence

Table 3 shows the significant associations between DV and support from the friends, being a member of any community group or organization and women’s attitude towards wife beating. Women who did not have the support of their friends were two times more likely to experience DV than women who had. Those women who were not members of any community group or organization were more likely to experience DV. Compared with women who did not accept that men are justified to beat their wives on any matter, those who accepted it partially and highly were approximately two times and three times more likely to report DV, respectively.

### Multivariate analysis

Finally, all variables found to be significantly associated in bivariate analysis were subjected to multivariate analysis. Variables such as the age of the husband, duration of the marriage, the number of living children, husband’s controlling behavior, and experience of violence before pregnancy were significant variables associated with DV. Among all the significant variables, women who experienced violence before the current pregnancy had the highest odds of experiencing DV. These pregnant women were 25 times more likely to experience DV (AOR = 24.55; 95% CI: 11.38–52.98) compared to women who did not experience violence before the pregnancy. As compared to those with husbands aged less than 25 years, women whose husbands were in the age group of 25–34 were 62% less likely to report DV (AOR = 0.38; 95% CI: 0.17–0.88). Also, a woman married for 2–5 years were 58% less likely to experience DV during pregnancy (AOR = 0.42; 95% CI: 0.20–0.90) compared to women who were married for less than and equal to 1 year. Women who had one or two children were 68% less likely to experience DV (AOR = 0.32; 95% CI: 0.11–0.88) compared to women who had no children. Likewise, DV was 1.9 times more likely to occur towards women who reported to experience at least one form of husbands’ controlling behavior (AOR = 1.88; 95% CI: 1.03–3.44), compared to those who had not experienced any of it (Table 5).

**Table 5:** Factors independently associated with domestic violence during pregnancy

Characteristics	*p* value	Adjusted OR
Age of husband		
Less than 25		Ref
25–34	0.023*	0.38 (0.17–0.88)
35 and above	0.150	0.40 (0.12–1.38)
Duration of marriage		
≤ 1 year		Ref
2–5 years	0.026*	0.42 (0.20–0.90)
6–9 years	0.956	0.97 (0.31–3.01)
10 and more years	0.848	0.86 (0.18–4.12)
Number of living children		
None		Ref
One or two children	0.027*	0.32 (0.11–0.88)
Husband’s controlling behaviour		
No		Ref
Yes	0.040*	1.88 (1.03–3.44)
Experience of violence before		
No		Ref
Yes	<0.001*	24.55 (11.38–52.98)

*Ref* = Reference

* = *p* value <0.05

## Discussion

The findings from the study showed that about one-fourth of the women (27.2%) experienced DV during the current pregnancy. A similar finding was seen in a study conducted in India (21%) [16] and in Mexico (25%) [17]. However, 38% of the women reported to have experienced DV during pregnancy [18] and almost half (44%) of the women reported having experienced abuse during the index pregnancy [19]. Different studies undertaken in different parts of the world showed fluctuating figures ranging from 4% in a study done in China [20] to 44% in Pakistan [19], thus supporting the fact that DV is very contextual and prevalence varies between different cultures and societies. In our study, sexual violence (17.3%) comprised the most common form of violence followed by psychological violence (16.6%) and the least common was physical violence (3.2%). The higher prevalence of sexual violence as compared to other studies may be due to the use of different definitions and measurement methods. In contrast to our findings, emotional abuse was the common form of the violence reported in most of the literature reviewed [21–26]. Consistent with our study findings, physical violence was seen to be lowest in studies conducted in Pakistan and Switzerland [24, 26].

When the pregnant women were asked whether they experienced violence before the current pregnancy, 20% percent of them reported having experienced some form of violence. The overall increase in cases of violence during the pregnancy could be due to the higher rates of sexual violence during pregnancy. The higher prevalence of sexual violence during pregnancy could be because most of the women may not have sexual desire during pregnancy. Moreover, our study did not measure any individual acts of physical, psychological and sexual violence before pregnancy. To ascertain which types of violence are present during pregnancy, and their changes in severity, further studies must be done. In contrast to our findings, almost half of the women (47%) reported some form of violence six months prior to their pregnancy while the prevalence of violence during pregnancy was 38% in a study undertaken in Pakistan [18]. About one-third of women reported that intimate partner violence reduced during pregnancy but the majority (69%) said that it either increased or remained same during pregnancy [27].

The husband was the main perpetrator in all types of violence with the husband being the sole perpetrator of both physical and sexual violence. This result is consistent with the study from Pakistan where the most common perpetrator of verbal, physical and/or sexual abuse was the woman’s husband, with the husband being the only perpetrator of sexual abuse and predominant perpetrator of physical abuse [18]. Comparable to our findings, most of the violence was perpetrated by a woman’s husband in many studies conducted in different parts of the world. Other perpetrators such as the mother-in-law, father-in-law, and sister-in-law committed very few DV towards women during pregnancy [20, 23, 25, 28].

Our study demonstrated that compared to women whose husbands were in age group of less than 25 years, those whose husbands were in the age group of 25–34 years were less likely to experience violence. A similar finding was seen where an increased partner’s age was found to be significantly associated with decreased odds of violence during pregnancy [17]. A possible explanation for this correlation is that when the husband’s age increases, he grows emotionally and socially and develops a sense of responsibility, thus leading to less interspousal conflict. However, in some studies, the husband’s age was not found to significantly affect the rate of DV during pregnancy [12, 21].

Compared to women married for less than one year, those married for more than 2–5 years were 57.7% less likely to experience DV during pregnancy, which indicates that the increase in years of marriage causes a decrease in DV. The positive association may be due to an increase in understanding between a husband and wife and the family members with time. The finding is comparable to the study conducted in an Iranian setting, where those in marriages of durations of 1–5 years and 6–10 years were more likely to experience psychological violence and physical violence than to women married for more than ten years [21]. In contrast, a study done in Karachi, Pakistan reported an increase in DV with an increase in duration of marriage [26].

The number of living children was significantly associated with the DV, with women who had one or two children less likely to suffer from DV than women who did not have any children. The probable justification may be that family members are reluctant to abuse women when their children are present. However, a study conducted in Pakistan showed an increased odds of violence with the increase in a number of children [19].

A study conducted in Thailand illustrated that pregnant women who were abused were more likely to be unemployed [29]. Likewise, unemployed women were more likely to experience violence during pregnancy as compared to employed women [16, 22]. However, our study showed no significant association between employment status and DV. This finding is consistent with the studies performed in various regions of the world [17, 19, 30]. Contrarily, a study conducted in slums in Mumbai reported that employed women were more likely to report violence than unemployed women [27].

The study illustrated that the odds of experiencing violence during pregnancy were approximately two times more likely among those women whose husband had control in any one of their wife’s activities. This finding is in line with other studies where strong positive associations were seen between husbands’ controlling behaviors and perpetration of violence against women [31–33]. Control plays an integral part in initiating the violence in the marital relationship. Violence due to husband’s controlling behavior has been regarded as patriarchal terrorism [32]. It is a result of a power imbalance where men consider themselves to be superior to their female counterpart. The association thus infers that there is still a presence of gender inequality and male dominance worldwide.

The history of DV was found to be the strongest predictor, with women who experienced some form of violence before pregnancy were approximately 25 times more likely to experience it during pregnancy. All women who reported DV during pregnancy had previously experienced some form of violence [22], and numerous studies have concluded violence before pregnancy to be strongly positively associated with DV during pregnancy [17, 28]. It proves that pregnancy does not protect women from being the victim of violence. Although there are contradictory thoughts of whether IPV initiates, increases or decreases during pregnancy, it was reported in a WHO multi-country study that most of the women who reported to have been abused physically were also beaten prior their pregnancy, whereas 50% of the women in three sites reported that they were beaten for first time during pregnancy [34].

Pregnancy is regarded as a socially and culturally respectful period of women’s life. Although there are several laws, policies and programs addressing violence against women, efforts to address violence specifically during pregnancy are still at an early stage. The Nepal Demographic Health Survey (NDHS, 2011) showed that 6% of the women who had ever been pregnant experienced physical violence. However, this national survey did not measure psychological and sexual violence. Despite the presence of management protocol for health service providers (2005) to address the violence, its use in the health service facilities is still in progress. Although studies exist on violence against women in Nepal, this is the first study to our knowledge addressing domestic violence amongst women during pregnancy.

### Limitations

The present study had some limitations. Since it was a cross-sectional study, the cause-effect relationship could not be established. Although the researcher had been trained adequately in rapport building and interviewing, the prevalence of DV may have been underreported due to the sensitive nature of the issue. Because the pregnant women were interviewed in the third trimester, experiences of violence after the interview of those pregnant women attending the ANC in the early period of the third trimester could not be detected. Our study sample consisted of only those pregnant women visiting the ANC. Hence the findings should not be generalized to cover all pregnant women in Nepal.

## Conclusion

The study demonstrated that DV was common among pregnant women attending antenatal clinics. The health care provider should utilize the opportunity of antenatal care to identify those women suffering from violence and provide required services to them by networking with other service providers. Several training programs providing in-service education to the health care professionals assessing the pregnant women are recommended. Routine screening with a structured questionnaire during ANC visits may help to diagnose DV instances among the pregnant women and prevent them from adverse health consequences. Furthermore, the fact that the history of violence was found to be the strongest predictor must be carefully taken into consideration by the health care providers. Because the study identified the husband as the main perpetrator, various counseling and awareness programs for men focusing on the harmful consequences of violence during pregnancy is recommended. Since the study revealed that controlling behavior from the husband was significantly associated with DV, programs focusing on women’s empowerment and providing vocational training to make them economically independent is of prime importance.

## Abbreviations

AOR: Adjusted odds ratio
CI: Confidence interval
DV: Domestic violence
IPV: Intimate partner violence
TUTH: Tribhuvan University Teaching Hospital
WHO: World Health Organization
